# A Preliminary Study of Gut Microbiota in Airline Pilots: Comparison With Construction Workers and Fitness Instructors

**DOI:** 10.7759/cureus.39841

**Published:** 2023-06-01

**Authors:** Piercarlo Minoretti, Camilla Sigurtà, Anna Fachinetti, Emanuele Cerone, Fabio Rotta, Enzo Emanuele

**Affiliations:** 1 Aviation Medicine, Studio Minoretti, Oggiono, ITA; 2 Aviation Medicine, Cavok Medical Center, Lonate Pozzolo, ITA; 3 Occupational Health, 2E Science, Robbio, ITA

**Keywords:** fitness instructors, construction workers, airline pilots, occupational medicine, gut, microbiota

## Abstract

Introduction: The term “WORKbiota” has been used to describe the impact of occupational exposure and work types on human microbiota composition. Airline pilots, construction workers, and fitness instructors encompass three diverse professional groups, each with distinct work environments and lifestyle factors that may significantly influence their intestinal “WORKbiota.”

Objectives: The current preliminary investigation was aimed to compare the relative abundance of specific gut microbes among airline pilots, construction workers, and fitness instructors to shed light on any significant differences. By scrutinizing these diverse professional groups, our objective was to enhance our understanding of how occupational factors influence gut microbiota while identifying possible implications for occupational medicine.

Methods: A convenience sample consisting of 60 men representing three different professional domains - airline pilots, construction workers, and fitness instructors (with 20 individuals in each group) - was selected during regular outpatient occupational health consultations. The abundance of selected gut microbiota constituents, including *Escherichia coli*, *Methanobrevibacter smithii*, *Akkermansia muciniphila*, *Faecalibacterium prausnitzii*, *Lactobacillus* spp., *Bifidobacterium* spp., and *Bacteroides* spp., was quantified using quantitative SYBR Green quantitative real-time polymerase chain reaction (qRT-PCR) in stool samples.

Results: There were no significant variations among the groups concerning *Escherichia coli*, *Methanobrevibacter smithii*, *Bifidobacterium* spp., and *Bacteroides *spp. However, *Lactobacillus* spp. and *Faecalibacterium prausnitzii* were significantly more abundant in the microbiota of fitness instructors compared to both airline pilots and construction workers, with no significant differences observed between the latter two groups. Notably, the abundance of *Akkermansia muciniphila* demonstrated a progressive decline from fitness instructors to construction workers and ultimately to airline pilots, who exhibited the lowest levels.

Conclusion: Airline pilots’ gut microbiota was characterized by a lower abundance of health-promoting bacterial species, including *Lactobacillus* spp., *Faecalibacterium prausnitzii*, and *Akkermansia muciniphila*. Future research is essential to determine whether targeted interventions, such as probiotic and prebiotic supplementation, could potentially enhance gut microbiota composition and overall health in particular occupational groups.

## Introduction

The study of the human gut microbiota and its connection to individual health has garnered significant attention in recent years [1,2]. This heightened interest is largely driven by the mounting body of evidence that highlights the impact of microbiota modifications on the development and progression of a wide array of disease conditions [3,4]. Considering that individuals devote a considerable fraction of their lives to occupational activities, it is reasonable to anticipate that work-related exposures and lifestyle factors will have a significant impact on the composition and diversity of an individual’s microbiota. In this context, Mucci et al. [5] have recently coined the term “WORKbiota” to describe the impact of occupational exposure and work types on human microbiota composition. Their literature review suggested that work-related modifications in microbiota could arise from multiple factors, including exposure to specific biohazards (such as those faced by agricultural and healthcare professionals), changes in dietary habits, prolonged contact with specific chemical substances, high-stress settings, distinctive microclimates, or prolonged journeys [5].

Airline pilots, construction workers, and fitness instructors encompass three diverse professional groups, each with distinct work environments and lifestyle factors that may significantly influence their intestinal “WORKbiota.” As an evolutionary land-adapted mammal, humans face unique challenges in aviation, which can negatively impact their health [6,7]. Airline pilots experience circadian disruptions due to shift work and irregular flight schedules [8], fatigue perception [9], cosmic ionizing radiation exposure [10], inconsistent meal times [11], mental stress [12], sedentary job nature [13], and cabin environment factors such as vibration, noise, and air quality [14]. Studies have also suggested an increased incidence of melanoma [15] and cardiovascular disease [16] among pilots compared to the general population, along with prevalent risks of low back pain, poor sleep, and mental health-related issues [6]. In contrast, construction workers face the challenges of physically strenuous tasks, encounter harmful environmental contaminants, and grapple with pervasive dust exposure [17,18]. On the other hand, fitness instructors typically partake in consistent physical exercise and frequently embrace more salubrious lifestyles [19], which may positively influence their gut microbiota composition.

The current preliminary investigation aimed to compare the relative abundance of specific gut microbes among airline pilots, construction workers, and fitness instructors to shed light on any significant differences. By scrutinizing these diverse professional groups, our objective was to enhance our understanding of how occupational factors influence gut microbiota while identifying possible implications for occupational medicine. Moreover, this study could aid in the development of customized interventions to strengthen gut health among individuals from diverse professions, ultimately boosting their job performance and overall well-being.

## Materials and methods

Study population

A convenience sample consisting of 60 men representing three different professional domains - airline pilots, construction workers, and fitness instructors (with 20 individuals in each group) - was selected during regular outpatient occupational health consultations. Women were not considered because the sample was too small. We excluded individuals with autoimmune, inflammatory, or infectious diseases, malignancies, or those who had received antibiotic treatment within three months prior to participating in the study. Furthermore, none of the participants were consuming probiotic supplements, and all appeared to be in good physical health. This study was approved by the local ethics committee (identifier: 2022/12) and all participants provided written informed consent.

Stool sample processing and extraction

The stool samples obtained from the study participants were preserved in sterile, screw-cap containers at a temperature of -70°C following collection. To isolate the total bacterial DNA, the QIAamp® DNA Stool Mini Kit (Qiagen, Hilden, Germany) was employed in accordance with the manufacturer’s instructions. A spectrophotometer was then utilized to accurately determine both the concentration and purity of the extracted DNA.

Quantitative real-time polymerase chain reaction

The abundance of selected gut microbiota constituents, including *Escherichia coli, Methanobrevibacter smithii, Akkermansia muciniphila, Faecalibacterium prausnitzii,* *Lactobacillus *spp., *Bifidobacterium* spp., and *Bacteroides* spp., was quantified using quantitative SYBR Green quantitative real-time polymerase chain reaction (qRT-PCR) [20]. The analysis was performed with a 7500 Fast Real-Time PCR System (Applied Biosystems, Foster City, CA) using specific primers (Table 1) [20,21]. The polymerase chain reaction (PCR) mixture and serial DNA dilution were prepared according to the established protocol [20]. Amplification reactions were carried out in a total volume of 20 μL, with the standard reaction mixture consisting of 10 μL SYBR Green PCR master mix (SensiFAST SYBR® Cat. No Bio-92020), 0.8 μL of each specific primer, and 2 μL of template DNA at a final concentration of 20 ng/μL. For *Akkermansia muciniphila*, the reaction mixture was modified to include 12.5 μL SYBR Green, 1 μL of each primer, and 2.5 μL of template DNA, resulting in a total reaction volume of 25 μL. In the negative control, 2 μL of sterile distilled H2O replaced the template DNA solution. The PCR conditions were set as follows: an initial denaturation at 95°C for five minutes, followed by 40 cycles of denaturation at 95°C for 15 seconds, primer annealing at a 50-70°C gradient for 20 seconds, and primer extension at 72°C for 45 seconds. A final extension step was performed at 72°C for five minutes. Fluorescent products were detected at the end of each cycle, and a melting curve analysis was conducted after amplification to differentiate targeted PCR products from non-targeted PCR products.

**Table 1:** List of primers used for investigating the abundance of selected gut microbiota constituents

	Forward primer (5’-3’)	Reverse primer (5’-3’)	Reference
Escherichia coli	GTTAATACCTTTGCTCATTGA	ACCAGGGTATCTAATCCTGTT	[21]
Methanobrevibacter smithii	CCGGGTATCTAATCCGGTTC	CTCCCAGGGTAGAGGTGAAA	[20]
Akkermansia muciniphila	CAGCACGTGAAGGTGGGGAC	CCTTGCGGTTGGCTTCAGAT	[20]
Faecalibacterium prausnitzii	AGATGGCCTCGCGTCCGA	CCGAAGACCTTCTTCCTCC	[21]
Lactobacillus spp.	GCAGCAGTAGGGAATCTTCCA	GCATTYCACCGCTACACATG	[21]
Bifidobacterium spp.	AGGGTTCGATTCTGCTCAG	CATCCGGCATTACCACCC	[21]
Bacteroides spp.	GTCAGTTGTGAAAGTTTGC	CAATCGGGAGTTCTTCGTG	[21]

Determination of absolute concentrations for selected gut microbiota constituents

The absolute concentrations of specific gut microbiota constituents were ascertained using a previously established method [20]. Standard curves were generated by employing 10-fold serial dilutions of reference strain genomic bacterial DNA with known concentrations. Colony-forming units were plotted against their corresponding cycle threshold (Ct) values. To determine the DNA microbiota constituents in the samples, the Ct values acquired from these samples were interpolated onto the relevant standard calibration curve.

Statistical analysis

Data are presented using descriptive statistics. To compare continuous variables among the three study groups, a one-way analysis of variance (ANOVA) was conducted, followed by a post hoc Tukey test for multiple comparisons. The chi-square test was utilized to analyze categorical variables. All statistical analyses were carried out using SPSS version 20.0 (IBM Corp., Armonk, NY). A two-tailed p-value <0.05 was considered statistically significant.

## Results

The three study groups exhibited no significant differences in age, body mass index, total cholesterol, and fasting plasma glucose (Table 2).

**Table 2:** General characteristics of the study participants Abbreviation: ns, not significant.

Variable	Airline pilots (n = 20)	Construction workers (n = 20)	Fitness instructors (n = 20)	P-value
Men	20	20	20	ns
Age, years	39.2 ± 3.3	38.9 ± 3.4	38.1 ± 2.1	ns
Body mass index, kg/m^2^	23.8 ± 2.3	23.9 ± 2.3	23.4 ± 1.6	ns
Total cholesterol, mg/dL	204 ± 9	209 ± 8	202 ± 10	ns
Fasting plasma glucose, mg/dL	89 ± 8	88 ± 10	90 ± 13	ns

An analysis of specific gut microbes (Table 3) revealed no significant variations among the groups concerning *Escherichia coli, Methanobrevibacter smithii, Bifidobacterium* spp., and *Bacteroides* spp. However, *Lactobacillus *spp. and *Faecalibacterium prausnitzii *were significantly more abundant in the microbiota of fitness instructors compared to both airline pilots and construction workers, with no significant differences observed between the latter two groups. Notably, the abundance of *Akkermansia muciniphila *demonstrated a progressive decline from fitness instructors to construction workers, and ultimately to airline pilots, who exhibited the lowest levels. The differences among each group proved to be statistically significant.

**Table 3:** Abundance of selected gut microbiota constituents in microbes among airline pilots, construction workers, and fitness instructors Data are expressed as copy numbers (mean ± standard deviation). * P < 0.05 versus fitness instructors; † P < 0.05 versus construction workers. Abbreviation: ns, not significant.

	Airline pilots (n = 20)	Construction workers (n = 20)	Fitness instructors (n = 20)	P-value
Escherichia coli	15.0 ± 5.3	14.8 ± 5.6	14.2 ± 4.7	ns
Methanobrevibacter smithii	7.6 ± 2.4	7.2 ± 2.7	7.5 ± 2.1	ns
Akkermansia muciniphila	4.4 ± 2.7*,†	5.2 ± 2.4*	6.4 ± 2.5	<0.001
Faecalibacterium prausnitzii	6.5 ± 2.5*	6.3 ± 2.7*	7.1 ± 3.2	<0.001
Lactobacillus spp.	9.1 ± 2.9*	8.9 ± 3.1*	10.7 ± 3.9	<0.001
Bifidobacterium spp.	9.6 ± 3.5	10.0 ± 3.7	9.9 ± 3.4	ns
Bacteroides spp.	8.0 ± 2.3	7.9 ± 2.1	7.7 ± 1.7	ns

## Discussion

Our investigation, which compared the gut “WORKbiota” among three distinct professional groups characterized by unique work environments and lifestyle factors, uncovered three major findings. Firstly, we found no significant differences between airline pilots, construction workers, and fitness instructors in terms of the abundance of *Escherichia coli*, *Methanobrevibacter smithii*, *Bifidobacterium* spp., and *Bacteroides *spp. This suggests that these bacterial species may not be significantly impacted by the varying occupational factors that distinguish the three professional categories. Secondly, the gut microbiota of fitness instructors exhibited a higher abundance of *Lactobacillus* spp. and *Faecalibacterium prausnitzii* in comparison to both airline pilots and construction workers, with the latter two groups demonstrating no significant differences between them. Finally, we observed a stepwise decrease in *Akkermansia muciniphila*’s abundance, starting with fitness instructors exhibiting the highest levels, followed by construction workers, and ultimately airline pilots, who displayed the lowest levels.

The connection between gut microbiota and physical activity has been well-documented [22], with research showing that specific beneficial bacteria, such as *Lactobacillus* spp., *Faecalibacterium prausnitzii*, and *Akkermansia muciniphila*, increase in response to exercise. Among these, commensal *Lactobacillus* spp. is considered the most crucial probiotic bacteria within the human gut microbiota [23]. They contribute to improved gut barrier integrity, enhanced mucosal barrier defense, and optimized host immune responses [24]. Furthermore, *Lactobacillus *spp. play vital roles in preventing the colonization of opportunistic pathogens in the gut [23]. These species achieve this effect by outcompeting harmful microorganisms for functional niches, inhibiting their attachment to the epithelium, and directly killing them through the production of lactic acid, propionic acid, acetic acid, and bacteriocins [25]. *Faecalibacterium prausnitzii* is the most abundant butyrate-producing bacterium found in human feces [26]. Numerous studies have highlighted the health-promoting properties of this microorganism, which are believed to be connected to its capacity to stabilize the intestinal mucosal layer, as well as reduce the activation of inflammatory pathways and generate butyrate [26]. In turn, butyrate has been shown to exert positive metabolic effects by preventing insulin resistance through epigenetic regulation, which enhances mitochondrial beta-oxidation, ultimately improving glucose sensitivity and reducing adiposity [27]. *Akkermansia muciniphila* is another beneficial bacterium that secretes enzymes into the intestinal tract, playing a crucial role in the local formation of mucin [28,29]. As a key constituent of the mucous layer that covers the gastrointestinal mucosa, mucin helps shield the intestinal lining from damage. When the mucous layer is compromised, it can lead to changes in intestinal permeability, a phenomenon commonly referred to as “leaky gut.” This condition has been linked to various disease conditions affecting not only the intestine but also the liver and brain [30].

Considering the well-established connection between an active lifestyle and a healthy human microbiota composition, it is unsurprising to find an increased abundance of *Lactobacillus* spp., *Faecalibacterium prausnitzii*, and *Akkermansia muciniphila* in fitness instructors. However, despite facing physically demanding tasks and showing a similar abundance of* Faecalibacterium prausnitzii *as fitness instructors, construction workers exhibited lower levels of *Akkermansia muciniphila*. This observation might be attributed to potentially healthier dietary habits among fitness instructors or specific environmental exposures to dust or chemicals. Interestingly, even with the different levels of physical activity typically exhibited by airline pilots and construction workers, there were no significant differences in the presence of *Lactobacillus* spp. and *Faecalibacterium prausnitzii *between the two professions. This observation indicates that elements beyond physical activity, including potential factors like disturbed circadian rhythms or heightened stress levels frequently experienced by pilots [6], might adversely influence the population of these advantageous gut bacteria.

The significant reduction of *Akkermansia muciniphila *in airline pilots, as compared to the other two groups, is remarkable. While our study does not allow us to deduce the underlying mechanisms, previous research on animals has demonstrated that ultraviolet irradiation can impact the fecal microbiome, including the abundance of* Akkermansia muciniphila* [31]. Consequently, the exposure of pilots to cosmic radiation [10] may play a significant role in the decrease of this beneficial species.

Our findings should be cautiously interpreted, considering several limitations. Firstly, the sample size was limited, necessitating further replication for validation. Furthermore, the study encountered a considerable challenge in addressing gender diversity. This can be primarily attributed to the underrepresentation of women in the construction workforce, which compelled us to focus exclusively on male participants. Secondly, the study did not provide insights into the mechanistic underpinnings behind the observed differences in gut microbiota composition among the three occupational groups. Additionally, dietary habits and physical activity measurements were not collected, as they were not included in the routine occupational medicine visits. Consequently, future research is essential to investigate the specific factors contributing to these differences and to determine whether targeted interventions, such as probiotic and prebiotic supplementation, could potentially enhance gut microbiota composition, quality of life, work performance, and overall health in particular occupational groups. Lastly, due to financial constraints, we opted to preselect specific gut microbes for analysis, rather than employing more expensive metagenomics techniques [32].

## Conclusions

This study provides, for the first time, valuable insights into the distinct gut “WORKbiota” composition of airline pilots, construction workers, and fitness instructors. The results not only corroborate but also broaden our understanding of the significant impact that occupational factors have on shaping an individual’s gut microbiota, which may hold crucial implications for their health. To gain a better grasp of the specific elements contributing to these disparities, additional research is warranted. This would also help identify potential interventions that could foster gut health and overall well-being across diverse occupational groups.
